# Cluster-randomized trial of monthly malaria prophylaxis versus focused screening and treatment: a study protocol to define malaria elimination strategies in Cambodia

**DOI:** 10.1186/s13063-018-2931-x

**Published:** 2018-10-16

**Authors:** Jessica Manning, Chanthap Lon, Michele Spring, Mariusz Wojnarski, Sok Somethy, Soklyda Chann, Panita Gosi, Kin Soveasna, Sabaithip Sriwichai, Worachet Kuntawunginn, Mark M Fukuda, Philip L Smith, Huy Rekol, Muth Sinoun, Mary So, Jessica Lin, Prom Satharath, David Saunders

**Affiliations:** 1 Laboratory of Malaria and Vector Research, Division of Intramural Research, National Institute of Allergy and Infectious Diseases, National Institutes of Health, Phnom Penh, Cambodia; 2 US Armed Forces Research Institute of Medical Sciences, Bangkok, Thailand; 3 Ministry of National Defense, Department of Health, Phnom Penh, Cambodia; 4 National Center for Parasitology, Entomology and Malaria Control, Phnom Penh, Cambodia; 5 University of North Carolina, Chapel Hill, NC USA

**Keywords:** Malaria, Elimination, Cambodia, Dihydroartemisinin-piperaquine, Primaquine, Permethrin, Mass drug administration

## Abstract

**Background:**

Malaria remains a critical public health problem in Southeast Asia despite intensive containment efforts. The continued spread of multi-drug-resistant *Plasmodium falciparum* has led to calls for malaria elimination on the Thai-Cambodian border. However, the optimal approach to elimination in difficult-to-reach border populations, such as the Military, remains unclear.

**Methods/design:**

A two-arm, cluster-randomized controlled, open-label pilot study is being conducted in military personnel and their families at focal endemic areas on the Thai-Cambodian border. The primary objective is to compare the effectiveness of monthly malaria prophylaxis (MMP) with dihydroartemisinin-piperaquine and weekly primaquine for 12 weeks compared with focused screening and treating (FSAT) following current Cambodian national treatment guidelines. Eight separate military encampments, making up approximately 1000 military personnel and their families, undergo randomization to the MMP or FSAT intervention for 3 months, with an additional 3 months’ follow-up. In addition, each treatment cluster of military personnel and civilians is also randomly assigned to receive either permethrin- or sham (water)-treated clothing in single-blind fashion. The primary endpoint is risk reduction for malaria infection in geographically distinct military encampments based on their treatment strategy. Monthly malaria screening in both arms is done via microscopy, PCR, and rapid diagnostic testing to compare both the accuracy and cost-effectiveness of diagnostic modalities to detect asymptomatic infection. Universal glucose-6-phosphate dehydrogenase (G6PD) deficiency screening is done at entry, comparing the results from a commercially available rapid diagnostic test, the fluorescence spot test, and quantitative testing for accuracy and cost-effectiveness. The comparative safety of the interventions chosen is also being evaluated.

**Discussion:**

Despite the apparent urgency, the key operational elements of proposed malaria elimination strategies in Southeast Asian mobile and migrant populations, including the Military, have yet to be rigorously tested in a well-controlled clinical study. Here, we present a protocol for the primary evaluation of two treatment paradigms – monthly malaria prophylaxis and focused screening and treatment – to achieve malaria elimination in a Cambodian military population. We will also assess the feasibility and incremental benefit of outdoor-biting vector intervention – permethrin-treated clothing. In the process, we aim to define the cost-effectiveness of the inputs required for success including a responsive information system, skilled human resource and laboratory infrastructure requirements, and quality management. Despite being a relatively low transmission area, the complexities of multi-drug-resistant malaria and the movement of vulnerable populations require an approach that is not only technically sound, but simple enough to be achievable.

**Trial registration:**

ClinicalTrials.gov, ID: NCT02653898. Registered on 13 January 2016.

**Electronic supplementary material:**

The online version of this article (10.1186/s13063-018-2931-x) contains supplementary material, which is available to authorized users.

## Background

Malaria remains a scourge to humankind with 270 million cases and over 600,000 deaths annually [1]. Despite intensive containment and control efforts, multi-drug-resistant (MDR) *Plasmodium falciparum* continues to be endemic in border areas of Cambodia. Over the last several years, reports have highlighted the declining efficacy of artemisinin combination therapies (ACT) within a few years of their introduction as first-line therapies [2–5]. As *P. falciparum* outpaces the development of efficacious antimalarial drugs, the focus has shifted to elimination of MDR malaria on the Thai-Cambodian border [4]. While the current means available to eliminate malaria are admittedly imperfect, waiting to act may result in a setback to recent gains in malaria control because of the increasingly rapid spread of resistance of *P. falciparum* and fewer treatment options being available [6].

To prevent the spread of multi-drug-resistant malaria, elimination is now being widely advocated in Cambodia, particularly in hard-to-reach mobile and migrant populations including the Military, an under-recognized reservoir of disease [2, 7]. Elimination of MDR malaria at the Thai-Cambodian border is critical given its transient populations who have the highest incidence of malaria in the Greater Mekong Subregion (GMS) [8, 9]. Harnessing the Military’s organizational capacity has recently been proposed as a novel strategy to effectively carry out malaria elimination programs in other more vulnerable mobile groups in close proximity along the borders [10, 11]. Such a strategy could be feasibly scaled as part of a national campaign, leveraging military infrastructure and organizational capacity, and emulated by other regional militaries in the GMS if successful.

However, the question remains: to effectively eliminate malaria in these populations, what are the optimal interventions? A potentially large, but as yet poorly defined, group of asymptomatic parasitemic persons poses a particular challenge to identifying the at-risk population [12–14]. Though more recent data in Cambodia suggests otherwise [15, 16], it has been presumed that submicroscopic asymptomatic parasitemias contribute significantly to ongoing transmission and thus constitute an infectious reservoir that must be addressed in low-endemic settings to achieve elimination [15, 17, 18]. If molecular diagnostic testing is needed to identify persons in this reservoir, or if medical follow-up requirements are too intense to support, large-scale implementation may be difficult or impossible in austere, remote locations. This conclusion has also been suggested by focused screen-and-treat programs as well as reactive case detection strategies in low-transmission areas of Cambodia in civilian populations [19–21].

Unfortunately, the only licensed, available drug able to prevent transmission of the parasite from human to mosquito, primaquine (PQ), can cause severe, life-threatening hemolytic anemia in persons with glucose-6-phosphate dehydrogenase (G6PD) deficiency, and requires effective screening tests that can be easily deployed at the point of care. To permit the greatest possible simplicity and cost-effectiveness during scale-up in resource-poor areas along the border, the ideal approach would require minimal medical monitoring or follow-up visits. Suitability for scale-up will also need to be assessed for operational feasibility, perception of risk among participants, cost and cost-effectiveness of interventions, and alignment with stakeholder values and policy.

### Aim and objectives

We present a pilot operational research protocol to investigate the effectiveness of monthly prophylactic drug administration compared to a focused screen-and-treat approach to inform a regional malaria elimination initiative by the Royal Cambodian Armed Forces (RCAF).

- *Primary objective*: to compare the effectiveness of focused screening and treatment (FSAT) following current national treatment guidelines versus monthly malaria prophylaxis (MMP) with dihydroartemisinin-piperaquine (DHA-PIP) in combination with weekly primaquine (PQ) (22.5 mg) as transmission-blocking and radical curative agent to reduce the risk of malaria infection in personnel residing in military encampments on the Thai-Cambodian border
- *Secondary objectives*:
  - ◦ To estimate the incremental benefit of wearing a permethrin insecticide-treated uniform (ITU) over drug therapy and existing vector control interventions compared to a sham insecticide-treated uniform (sITU) treated with water. Civilian participants will have comparable selected outer garments treated
  - ◦ To define the proportion of asymptomatic carriers of malaria in the population, including the cumulative incidence and incidence density of microscopic and submicroscopic blood-stage parasitemia and gametocytemia
  - ◦ Additional secondary objectives can be found in Table 1/Additional file 1

**Table 1:** Outcomes

Objectives	Outcomes/endpoints
Primary	
To compare the effectiveness of focused screening and treatment (FSAT) following current national treatment guidelines versus targeted monthly malaria prophylaxis (MMP) with DHA-piperaquine in combination with low-dose weekly primaquine (PQ) (22.5 mg) for 12 weeks	Absolute risk reduction in a cluster based on those individuals’ polymerase chain reaction (PCR)-corrected absence of parasitemia at the end of 6 months in the MMP versus FSAT clusters
Secondary	
Estimate the incremental benefit of insecticide-treated uniforms (ITU) over drug therapy and existing vector control interventions compared to a sham insecticide-treated uniform (sITU)^a^	Absolute risk reduction in a cluster based on those individuals’ PCR-corrected absence of parasitemia at the end of 6 months^a^ Proportional landings of mosquitoes on treated uniform’s swatch side of cages
Assess the effectiveness of presumptive anti-relapse and transmission-blocking therapy with weekly low-dose primaquine (22.5 mg)^a^	Incidence of *P. vivax* recurrent infection and PCR-corrected *P. falciparum* recrudescence^a^ PCR-corrected absence of gametocytemia^a^
Evaluate the safety and tolerability of blood-stage antimalarials and weekly low-dose primaquine at 12 weeks versus 8 weeks in treated MMP and FSAT volunteers, respectively	Number of hemolytic events or other serious adverse events in participants over 13 years of age receiving primaquine
Assess level of antimalarial drug resistance at the selected study sites	Number of all-species malaria recurrence, established molecular markers of drug resistance, and clinical failure rates based on WHO criteria
Define the proportion of asymptomatic carriers of malaria in the study population	Cumulative incidence and incidence density of PCR-corrected parasitemia and submicroscopic parasitemia in each arm
Define the epidemiology of malaria infection in volunteers developing malaria	Cumulative incidence and incidence density of parasitemia
Compare the sensitivity and specificity of the currently recommended malaria rapid diagnostic test of choice in Cambodia with RT-PCR	Retrospective assessment of the proportion of asymptomatic carriers that would have been missed by each test and an estimate of the incremental cost-effectiveness of each test
Compare sensitivity and specificity of two currently available rapid diagnostic tests to detect moderate to severe G6PD deficiency using quantitative G6PD testing as the reference standard	Retrospective assessment of the proportion of persons with moderate to severe deficiency who would have been missed with each screening modality, and an estimate of the cost-effectiveness of each test
Describe population demographics to include the acceptability of FSAT, malaria prophylaxis, and use of vector control measures among participants, including willingness to participate in future malaria elimination campaigns	Descriptive analysis of participants’ responses to survey questions pre and post study regarding FSAT, malaria prophylaxis, and use of vector control measures
Support host nation capabilities by improving the Royal Cambodian Armed Forces’ (RCAF’s) ability to diagnose, prevent, and treat malaria supported by robust data to achieve malaria elimination	A scalable military malaria elimination “unit of action” will be established at the provincial level, staffed by RCAF personnel trained during the course of the study

^a^Pilot study objective and endpoint added to assess operational feasibility given ongoing community concerns regarding these interventions. These endpoints are statistically underpowered and this data will be used to inform larger studies in the future

Lastly, an overarching aim of this operational study is to increase The Cambodian Military’s capability to diagnose, prevent, and treat malaria, and to understand the population’s willingness to participate in malaria elimination campaigns. To do so, a scalable military malaria elimination “unit of action” is being established at the provincial level, staffed by RCAF personnel who are trained during the course of this study. Regardless of the outcome, the study will provide critically important information to inform elimination policy in Cambodia and the GMS.

## Methods/design

### Study design

We are conducting a two-arm, cluster-randomized, open-label controlled trial of MMP with t3-day DHA-PIP and weekly PQ compared to focused screening and treating (FSAT) following current national treatment guidelines. Participants in the MMP arm receive a monthly 3-day course of fixed-combination tablet containing 40 mg of dihydroartemisinin and 320 mg of piperaquine phosphate for three consecutive months. Those aged 13 years and older receive low-dose weekly PQ (22.5 mg) therapy to prevent malaria transmission for 12 weeks. Participants in the FSAT arm are screened with highly sensitive polymerase chain reaction (PCR) methods, and all those testing positive for malaria receive therapy with first-line antimalarials following current national treatment guidelines. At the time of protocol development, artesunate-mefloquine combination therapy is the first-line treatment for *P. falciparum* in provinces with known antimalarial resistance, with a single, low dose of PQ (15 mg) to prevent malaria transmission. For participants in the FSAT arm with *Plasmodium vivax* malaria, the recommended treatment for asexual parasitemia is DHA-PIP. All patients with *P. vivax* in the FSAT arm receive treatment with 15 mg of PQ daily for 14 days if G6PD normal, or 45 mg weekly if G6PD deficient.

### Study site and population

The study takes place at multiple military encampments in malaria-endemic areas in border areas authorized by the Ministry of National Defense. Sites were chosen based on current malaria incidence estimates by the RCAF health services in consultation with the National Center for Parasitology, Entomology, and Malaria Control (CNM) in Cambodia and the Armed Forces Research Institute of Medical Sciences (AFRIMS). For reasons of operational security, locations or other descriptors will not be further described here. All sites will be evaluated as simultaneously as feasibly possible.

Military volunteers aged 18–65 years of age and their dependents of ≥ 2 years of age, eligible for care at an RCAF facility, or otherwise eligible Cambodian civilians at risk for contracting malaria who live within close proximity, are enrolled if they meet the following criteria: are able to give informed consent/assent; reside in the selected study areas; are available for monthly follow-up for 6 months; agree not to seek outside medical care for febrile illness unless referred by study team; and are authorized by respective military unit commanders to participate in the study if on active duty. Volunteers who have known allergy or other contraindication to study medication; are pregnant, lactating or are women of childbearing age who do not agree to use an acceptable form of contraception during the study; or are judged by the investigator to be otherwise unsuitable for study participation are excluded from the study.

### Cluster randomization, allocation, and blinding

Geographical areas were randomized prior to study start to a 1:1 allocation of MMP or FSAT using time- and region-blocked randomization to ensure that randomization is geographically distinct, and that malaria incidence is evenly distributed among groups given that high and low transmission areas exist. Volunteers meeting the enrollment criteria receive permethrin- or sham-treated (water-treated) uniforms according to their cluster’s assignment in single-blind fashion (volunteer blinded to assignment but not investigator). Entomology staff conducting uniform testing are also blinded to treatment. Diagnostic microscopists are blinded to each others’ readings and to study arm assignment. There is otherwise no blinding during the study.

### Treatment intervention

Volunteers randomized to MMP arm aged 13 years and older receive a monthly 3-day treatment course of (DHA-PIP; 360/2880 mg) for 3 months. They will also receive a weekly dose of 22.5 mg of PQ for 12 weeks. Children of 12 years of age or less will receive only a monthly 3-day weight-based treatment dose of DP for 3 months without PQ treatment. In the MMP arm, symptomatic patients or microscopy-confirmed parasitemia in the first 3 months are considered a failure of prophylaxis and treatment is given according to the national treatment guidelines. If the volunteer has parasitemia detectable by PCR only and is symptom free at monthly follow-up, the study treatment with monthly DHA-PIP is continued for 3 months as outlined for the MMP arm. To reduce phlebotomy requirements for G6PD testing and potential hemolytic risk in children, PQ is not be administered to those less than 13 years old.

Volunteers randomized to the focused screening and treatment (FSAT) arm are treated according to current national malaria treatment guidelines if they test positive for malaria based on blood smear and/or real-time PCR results during monthly follow-up. In the FSAT arm, all volunteers with parasitemia detectable with PCR receive antimalarial treatment, even if they are symptom- free.

**Fig. 1:**
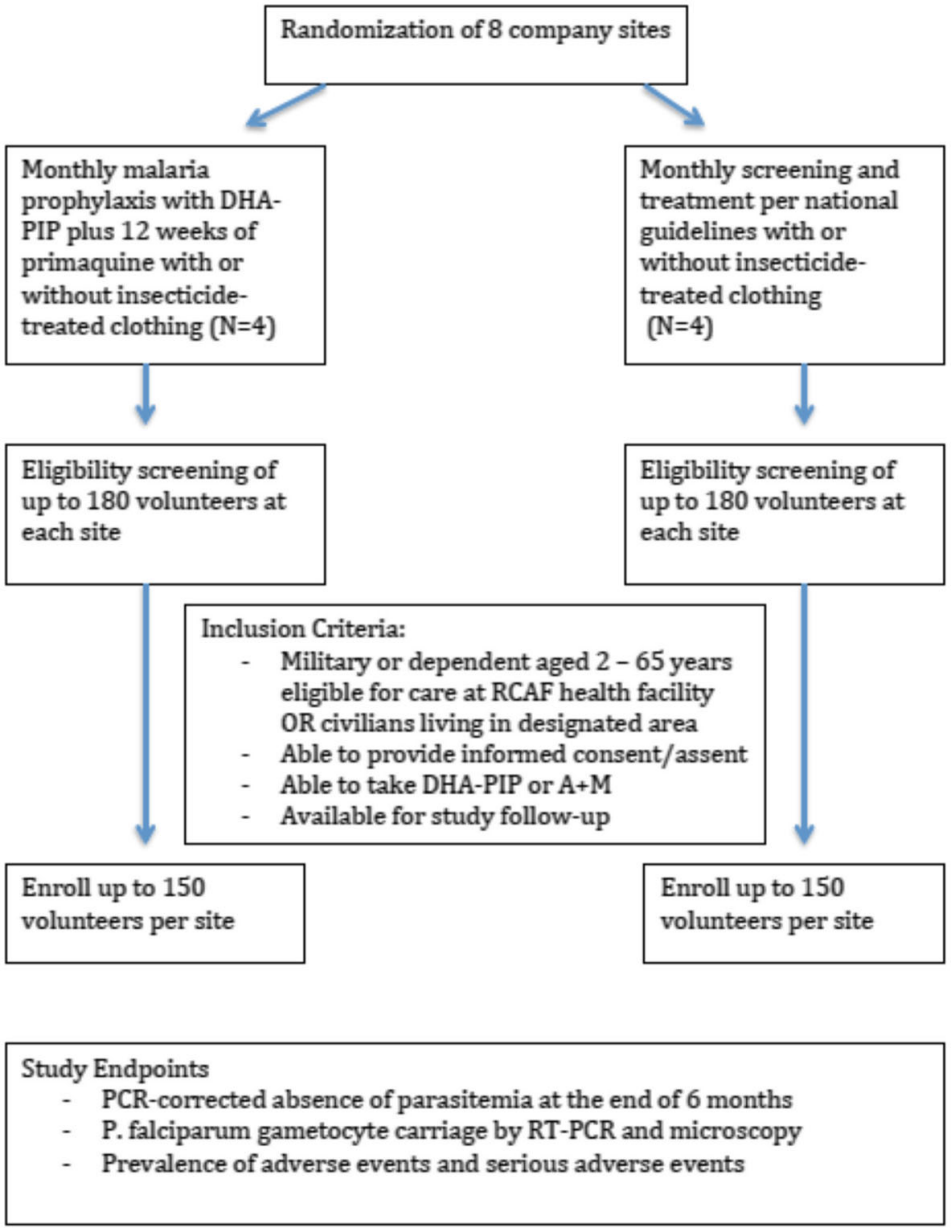
Study flow diagram

**Fig. 2.**
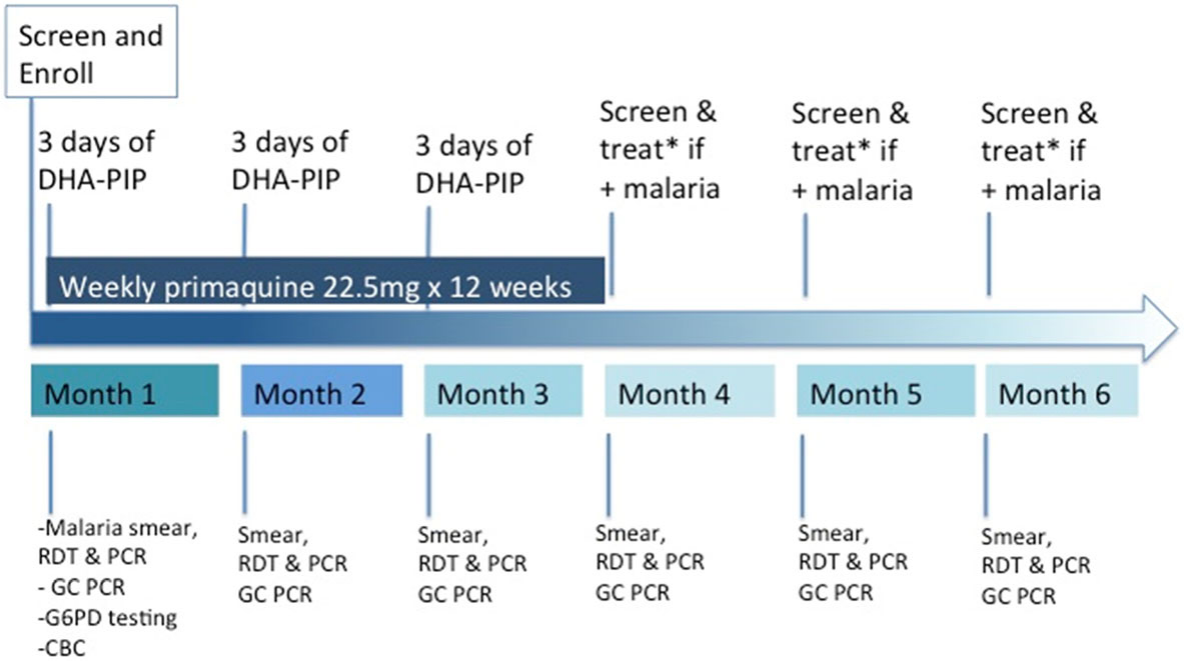
Study timeline for monthly malaria prophylaxis (MMP) intervention clusters. *Treat with artesunate and mefloquine. *DHA-PIP* dihydroartemisinin-piperaquine, *GC* gametocyte, *RDT* rapid diagnostic test, *PCR* polymerase chain reaction, *G6PD* glucose-6-phosphatase dehydrogenase, *CBC* complete blood count

At the time of protocol development, national treatment guidelines for areas of known piperaquine resistance, to include the designated study regions, recommended a 3-day course of artesunate and mefloquine (A + M) and a single 15-mg dose of PQ for acute *P. falciparum*, or DP for blood-stage *P. vivax* infection. Elimination interventions are carried out monthly during the first 3 months after enrollment, and then followed by 3 months of additional malaria assessment by smear and PCR diagnosis with malaria treatment as necessary following current national treatment guidelines (Fig. 1, Table 2, Fig. 2). Those febrile but negative for malaria are referred for evaluation and treatment of alternative diagnoses.

**Table 2:** Table of times and events for adults and children over 12 years of age

Event	Screen/enroll day 1	Day 3^3^	Day 30^7^	Day 60^7^	Day 90^7^	Day 120^7^	Day 150^7^	Day 180^7^	Malaria diagnosis/ recurrence^2^
a. Informed consent	X								
b. Medical history	X								
c. Physical exam	X								X
d. Brief clinical evaluation and vital signs^1^			X	X	X	X	X	X	X
e. Malaria RDT, DBS, smear and PCR genotyping^6^	2 mL		1.5	1.5	1.5	1.5	1.5	1.5	1.5 mL
i. CBC	1 mL	1 mL^3^							
k. Molecular resistance markers^2^									6 mL
l. G6PD RDT, fluorescent spot, quantitative testing	1.5 mL								
m. Gametocyte PCR	2.5 mL		2.5	2.5	2.5	2.5	2.5	2.5	2.5 mL
n. Urine pregnancy test (women of child-bearing age)	X		X	X					X
o. Malaria treatment^4,5^	X		X	X					X
Daily phlebotomy (mL)	7	1^3^	4	4	4	4	4	4	10
Cumulative phlebotomy (approximately mL)	7		11	15	19	23	27	31	41

^1^Brief clinical evaluation includes an interval medical history, vital signs and a directed physical exam as indicated

^2^Performed only for volunteers with smear-positive malaria. Those with PCR-positive malaria will be treated following national guidelines but will not have blood drawn for molecular markers of resistance

^3^To be drawn for G6PD-deficient volunteers only. All G6PD-deficient volunteers with > 10% of Hgb or HTC drop on D3 will have CBC on day 7

^4^Patients screening positive on microscopy and/or PCR for *P. falciparum* malaria in the FSAT arm will receive currently recommended blood-stage antimalarials under published national treatment guidelines as well as single, low-dose primaquine (15 mg). Volunteers with *P. vivax* will be treated with the currently recommended blood-stage agent (DHA-PIP), as well as primaquine – G6PD-normal volunteers will receive 15 mg daily for 14 days, while G6PD-deficient volunteer will receive 45 mg × 8 weeks. All volunteers in the MMP arm will receive a fixed-dose 3-day course of DHA-piperaquine at 0, 24 and 48 h starting on days 1, 30, and 60, and a weekly 22.5-mg dose of primaquine for 12 weeks. All therapy will be directly observed

^5^For all volunteers with recurrent malaria, rescue therapy will be with the recommended agent(s) per national guidelines

^6^May be repeated as medically indicated (e.g., if patient is malaria-positive or otherwise ill on enrollment). An additional 0.5 mL will be collected on the day of enrollment for baseline G6PD genotyping

^7^Time window of ± 7 days

*CBC* complete blood count, *DBS* dried blood spot, *DHA-PIP* dihydroartemisinin-piperaquine, *G6PD* glucose-6-phosphatase dehydrogenase, *Hgb* hemoglobin, *PCR* polymerase chain reaction, *RDT* rapid diagnostic test

### Clinical procedures

Following documentation of informed consent/assent and determination of eligibility, a study physician completes an initial medical history and physical examination (Table 2). Volunteers aged 13 years and older have a rapid diagnostic test (RDT) for malaria, thick and thin blood films to estimate both sexual and asexual parasite densities, and malaria PCR diagnosis. Additional laboratory testing includes a complete blood count (CBC); an estimate of G6PD activity using quantitative testing to be compared with fluorescence and commercially available point-of-care testing; G6PD genotyping; real-time PCR to detect gametocytes; and analysis of molecular markers of resistance for those malaria positive at intake. Children aged 12 years and under have only rapid malaria diagnostic testing, malaria smears and PCR to detect submicroscopic parasitemia, with other testing deferred in children to reduce phlebotomy burden. Children less than 13 years of age are not tested for G6PD deficiency as they are not treated with PQ under this protocol. G6PD-deficient volunteers 13 years and older are counseled regarding the diagnosis, and have a repeat CBC on day 3 (± 1 day) following PQ treatment with repeat testing on day 7, if they have a greater than 10% drop in their hemoglobin or hematocrit, or at any time there are clinical signs of hemolysis.

All volunteers are followed monthly thereafter or at any time that they present with symptoms of suspected malaria, with a brief clinical evaluation and malaria diagnostics to include PCR assays for asexual and sexual parasites. Malaria-positive volunteers aged 13 years and older have additional tests for molecular markers of resistance. At these times, a swatch is taken from the volunteer’s uniform or clothing for repellency monitoring.

At the conclusion of the 180-day (− 2 to + 3 days) follow-up period, volunteers have blood drawn for malaria smears, RDTs, asexual blood-stage and gametocyte PCR diagnosis. If infection is noted at discharge, volunteers will continue to be followed until resolution of infection.

### Laboratory procedures

#### Microscopy

Stained thick and thin blood smears are examined by two microscopists blinded to each other’s results and to the treatment status of the study volunteer. Two blood smears are made for every enrolled volunteer. Slide 1 is stained immediately and examination of Giemsa-stained thick and thin smears made. This slide is stored in a different box from slide 2, which is only read if there is a problem with the first slide.

Parasite densities are calculated based on a count of parasites per 200 white blood cells (WBCs) (thick film) or for low parasitemias (e.g., 10 parasites/μL), per 500 WBC or 5000 red blood cells (RBCs) (thin film). Both asexual and sexual stages are enumerated. A total of 200 oil immersion fields are examined on the thick film before a blood smear is considered negative. The final count is determined by taking the geometric mean of the two counts. In case of a difference in results (positive/negative; species diagnosis) between the two microscopists, the blood smear is re-examined by a third microscopist blinded to the results of the first two readers and the treatment regimen, and the third reading is accepted as the final result.

Malaria microscopy results is confirmed using real-time PCR correction for the detection of *P. falciparum* and *P. vivax* using 18S ribosomal ribonucleic acid (RNA) (*18S rRNA*) genes unique to each species. Parasite deoxyribonucleic acid (DNA) will be isolated from approximately 200–250 μL of venous or capillary blood collected in an EDTA microtube.

#### PCR genotyping

One milliliter of blood is drawn on day 1 if malaria-positive and at recurrence. PCR genotyping of *msp1*, *msp2*, and *GLURP* genes is performed to identify the unique fingerprint of the infecting parasite and any subsequent development of malaria after therapy in order to determine if it is a recrudescent infection or new infecting genotype [22]. Gametocyte PCR and molecular marker analysis is performed as previously described [23–26].

#### G6PD testing

On enrollment, volunteers are assessed for G6PD deficiency with both fluorescence (qualitative) and quantitative testing, and one or more commercially available point-of-care test kits, and correlated with single nucleotide polymorphism analysis for G6PD gene mutations. Approximately 1.5 mL per blood draw is collected in an EDTA (anticoagulant) tube for this purpose.

Venous blood is tested for qualitative G6PD activity using the fluorescent spot test method, as recommended by the International Committee for Standardization in Hematology using commercially available kits (R&D Diagnostics Ltd., Greece). This method detects fluorescence of the enzyme NADPH under long-wave (365 nm) UV light. Reduction of NADP to NADPH occurs in the presence of G6PD. The rate and extent of NADPH formation is proportional to G6PD activity. Normal samples fluoresce brightly, whereas deficient samples show little or no fluorescence [2].

Quantitative testing will be performed using an FDA-approved test kit (e.g., Trinity Biotech, Ireland) and results will be calculated based on same-day hemoglobin values from the CBC. Severe deficiency (WHO class I or II) will be defined as 10% or less of the lower limit of normal activity (in G6PD activity units per gram of hemoglobin) established for the quantitative assay system. Subjects with severe deficiency will be enrolled in the study as this is not an exclusion criterion; class I–V deficiencies are permissible for enrollment. To evaluate the performance of a commercial G6PD deficiency RDT in malaria patients from Cambodia, the CareStart™ G6PD test is being compared to the “gold standard” G6PD enzyme quantitative test for their potential use as point-of-care diagnostics for G6PD deficiency. However, no clinical decisions will be made based on the results of either point-of-care test in this study.

#### Permethrin clothing treatment

Each uniform is treated by study staff according to the site’s randomization prior to distribution with a single application of the 40% permethrin Individual Dynamic Absorption (IDA) kit from the US Army’s supply system or water only for sham-treated uniforms. Permethrin treatment is designed to last for 30 washings, which should be suitable for the 6-month duration of the study. At enrollment, each volunteer has one to two field uniforms treated, and civilian volunteers have one to two sets of outer covering garments that they wear while in forested areas treated. The number of clothing sets treated will depend on the number in the volunteer’s possession, and additional clothing sets may be treated during the course of the study if/when the volunteer acquires new clothing, or when treated clothing is damaged or becomes otherwise unserviceable.

Monitoring of treated uniform repellency will be performed by taking uniform swatches numbered with the subject’s study ID at monthly intervals for 6 months. The numbered swatches will be sewn into the uniforms at the time of treatment. Blinded entomology technicians will randomly select 5% of uniform swatches, and place them in cages of female mosquitoes to observe for repellency. The clothing swatches will be placed on the wall of the mosquito cages and proportional landings on the swatch side of the cage will be assessed.

### Sample size rationale

Up to 1200 persons are being enrolled and followed in this cluster-randomized study. The study compares estimates of the absolute risk reduction based on the proportion remaining malaria free at the end of 6 months. We assume that MMP administration combined with an insecticide-treated uniform (ITU) on the background of any pre-existing vector control methods that might be employed will need to reduce malaria incidence to zero by study conclusion to achieve elimination. The MMP approach will be using nearly every currently available intervention in Cambodia to eliminate malaria in this population. Current mathematical models show that multi-round mass drug administration (MDA) can reduce malaria prevalence to less than 10% if high coverage is achieved [27]. Focused screening and treatment alone (FSAT) is assumed to be 50% effective based on a recent study at the same site which found that only 50% of patients treated using an intense screening and treatment approach remained malaria free at the end of 4–6 months of follow-up [2]. The study is, therefore, powered to detect a two-fold difference in effectiveness between the two intervention groups. These are thought to be reasonable assumptions a priori for the purposes of sample size calculations though there has never been a head-to-head comparison of these strategies published in the literature. Given that this will be a stratified analysis, no assumptions regarding vector control measures are included in the calculation. Pre-existing vector control measures that the Military may employ, including ITNs, are assumed to be equally distributed among the overall study population. Permethrin-treated uniform comparisons are a secondary endpoint intended only to estimate a potential additive effect.

Because observations of individuals in the same geographical area (e.g., cluster) are correlated (non-independent), the effective sample size needed is larger than an individualized randomized trial. Given that the intracorrelation coefficient (ICC) is not known, we assumed a conservative value of 0.4. To detect a minimum of a two-fold difference in the effectiveness of MDA over FSAT in proportion of volunteers being malaria-free at 6 months with an α = 0.05 and 80% power, the number needed in each cluster is at least 78 individuals [28]. Given that the number of clusters is limited based on RCAF organization and patrolling areas, our proposed cluster number of 8 with a size of 80–140 volunteers per cluster would be sufficient even at higher (e.g., more conservative) values of ICC (> 0.4).

### Statistical methods

The primary study endpoint will be the absolute risk reduction (ARR) with 95% confidence intervals (CIs) based on the proportion of volunteers in each arm with absence of PCR-positive parasitemia at the end of 6 months using an intention-to-treat analysis (Table 1). In addition, logistic regression modeling may be employed to provide multivariate analyses based on other possible study confounders such as age, proportion of children, proportion of pregnant women, etc. The primary study population will be intention-to-treat, defined as all volunteers (both military and civilian family members) who are enrolled at either site. The per-protocol analysis population will include all those volunteers who enrolled and completed follow-up up to 180 days. However, volunteers lost to follow-up that do not complete up to 180 days of follow-up will be excluded from the per-protocol analysis, but included in an intention-to-treat analysis.

Secondary study endpoints are pilot endpoints to include incidence rates, molecular and parasitological endpoints, safety, vector control effectiveness (e.g., permethrin-treated versus water-treated uniforms), and comparison of malaria and G6PD diagnostic modalities. The calculation of incidence rates (attack rate over total time at risk) for prophylactic and FSAT groups will be calculated adjusted and unadjusted with 95% CI. All volunteers completing at least one follow-up assessment will be included in the safety analysis. The safety analysis database will include those volunteers in the set of randomized volunteers who receive at least one dose of study drug. All parasitological data will be included in the parasitological analyses. A stratified analysis comparing the vector control arms within each group will also be compared as those remaining malaria-free at 180 days. Given the possibility of the highly variable nature of transmission at the geographical sites, existing pre-enrollment malaria surveillance data by RCAF and day 1 screening data by AFRIMS will guide if a pair-matched analysis is necessary.

### Halting rules criteria

Any volunteer with G6PD deficiency who experiences grade 3 hemolysis following PQ administration will be discontinued from the use of PQ and monitored closely. If more than three subjects with G6PD deficiency are found to have grade 3 hemolysis following treatment with PQ for anti-relapse therapy, further treatment with PQ will be suspended for all G6PD-deficient subjects enrolled in the study.

### Data storage and handling

Clinical and laboratory data pertaining to drug efficacy are collected and managed by AFRIMS Immunology and Medicine staff and RCAF personnel. All data and medical information obtained from study volunteers are considered privileged and confidential. Volunteers enrolling in the study are issued a unique identification code (UIC), which is used on all study files and clinical sample labels. Individually identifiable volunteer information other than the UIC are not transcribed on other study documents to include laboratory sample labels, case report forms, nor included in the presentation of study results. Data is double-entered into a Microsoft Access database (Microsoft Corp) with source data verification for each entry.

### Ethical approval

This trial has been approved by the Walter Reed Army Institute of Research (WRAIR) and the National Ethical Committee for Health Research (NECHR) in Cambodia.

## Discussion

### Overall rationale for the study

Elimination of malaria in mobile and migrant populations in border areas is of increasing importance as these transient populations carry a significant amount of MDR malaria burden in the GMS [8, 29, 30]. Despite the apparent urgency and attention to defining Southeast Asian mobile and migrant populations [31], the key operational elements of proposed malaria elimination strategies in these groups have yet to be rigorously tested in a well-controlled clinical study. This operational study protocol aims to define the most effective and reproducible components of a malaria elimination program specifically among Cambodian military forces. While the choice of approach and clinical regimen are important and oft-debated, the underlying premise of the present study is that the active engagement of military forces is likely to have a far reaching impact on overall elimination efforts. The sheer size, reach, and logistical capabilities of military forces leave them ideally suited to conduct large-scale elimination campaigns [11]. In that sense, the study also serves as a real-life training exercise for Cambodia’s military forces to develop needed capacities to manage malaria elimination efforts. The challenges of conducting a well-controlled elimination study are the same as those of conducting malaria elimination itself. Activities must be carefully coordinated by health workers trained in the use of exacting standard operating procedures and documented flawlessly. Functioning laboratories capable of maintaining high-quality control standards must be established. Much like military campaigns, these intensive efforts must be sustained for months or years. On all of these counts, the present study serves as the ideal training ground to build capacity and the skilled workforce that will be needed for Cambodia to scale up rigorous elimination efforts.

### Challenges in malaria elimination

While terms describing “mobile populations” are often used in reference to malaria elimination, they remain poorly defined, and encompass a wide range of persons including economic migrants, ethnic minorities, military and other government personnel, and tourists. Mobility among malaria endemic areas is their common shared trait, and for the most part, their mobility makes them more difficult to study. In this regard, military and government personnel may be the easiest of these varied groups to study because of the ability to provide comprehensive malaria prevention and treatment services reliably despite frequent movement. Military organization and chain of command provide frequent opportunities for structured intervention, suggesting that militaries may be among the easiest mobile groups to achieve early malaria elimination. Despite these advantages, the need to preserve operational and information security and the need to ensure that research does not interfere with the imperatives of military service remain important barriers. The Military is also considered a vulnerable population for medical research, and additional steps must be taken to ensure that study participation is never coerced through the chain of command. Potential transmission between surrounding communities and frequent personnel movements are additional challenges in designing interventions and assessing results [11].

### Comparison of MMP versus FSAT, and problems with the term “MDA”

The study is effectively comparing a large-scale malaria prophylaxis approach using currently available interventions with a more systematic version of current public health efforts to identify and treat infected persons. The primary objective of this study is to directly compare the effectiveness of MMP with DHA-PIP and weekly PQ against an active screening and treatment strategy following the current national malaria treatment guidelines. In the province where the study was conducted at the time of study inception, these included artesunate and mefloquine combination treatment for all acutely infected persons with *P. falciparum* malaria and DHA-PIP for *P. vivax* malaria. While PQ use – either single-dose for *P. falciparum* transmission-blocking or 14-day anti-relapse therapy for *P. vivax* infections – is currently advocated by treatment guidelines, implementation has been limited and use officially restricted to facilities able to screen for G6PD deficiency, greatly limiting application outside controlled research studies. Perhaps more important is the question of which, if any of the drugs involved can be safely administered on a large scale, with minimal or no medical supervision. Such practice, termed “mass drug administration,” is often considered as a means to treat significant numbers of infected individuals in limited-resource settings [32]. Because this term can be misconstrued, we have termed the key study intervention “malaria prophylaxis” rather than “mass drug administration” – the purpose is not merely to eradicate malaria within the individual, but to protect all treated individuals and their compatriots, treated and untreated, from the collective threat of malaria transmission. While blood-stage agents, such as A + M and DHA-piperaquine, achieve the former, only PQ administration has been shown to treat mature gametocytes responsible for mosquito transmission [33].

### Selection of safe, effective prophylaxis from a dearth of options

Selection of appropriate chemoprophylaxis requires a safe, tolerable, and effective drug that can be administered widely in the population. Such options, limited to begin with, are rapidly dwindling in the GMS as MDR malaria continues to gain a greater foothold [6]. The investigators selected DHA-PIP despite recent reports of declining treatment efficacy [3], though this was by no means a straightforward decision. Factors favoring its use included prophylaxis studies demonstrating efficacy of monthly administration of full DHA-PIP treatment courses in settings of known MDR malaria [34–36]. DHA-PIP remains inexpensive and very well-tolerated with an excellent safety profile, making it an ideal candidate for widespread administration [37, 38]. QT-interval prolongation is a known effect of piperaquine administration, though the clinical significance remains unclear, and limited data suggests reduced risk with a standard 3-day course of therapy [39]. Atovaquone-proguanil was also considered, but given its expense, daily administration and potential for rapidly developing resistance, the requirement for directly observed therapy (DOT) and strict follow-up would be difficult to scale up. Furthermore, mathematical modeling suggests that atovaquone-proguanil for MDA would result in further spread of resistance with only temporary returns in elimination of malaria [40]. Lastly, a newer ACT, pyronaridine-artesunate has been found to be well-tolerated and non-inferior to artemether-lumenfantrine and artesunate-mefloquine, but limited safety data precludes its use for an elimination campaign [41–43]. Though far from ideal, DHA-piperaquine was selected as the *least* unfavorable of currently available options in order to make current progress toward elimination rather than await alternatives. Though its efficacy has plummeted below accepted thresholds for treatment of acute *P. falciparum* infection, it is likely to retain efficacy against subclinical *P. falciparum* encountered in pre-elimination settings, and *P. vivax* infections [2, 3, 13, 44].

### Primaquine and transmission-blocking therapies

While considered an essential component for malaria elimination [45–47], the safety of PQ must be given careful consideration given the risk of hemolysis in G6PD-deficient individuals, who may be at even more risk in a large-scale elimination campaign that does not allow for close, individual follow-up [48]. Grimmond et al. demonstrated safe use of 22.5 mg of PQ weekly in combination with chloroquine for 8 weeks to prevent *P. vivax* relapse in a cohort of Southeast Asian refugees in Australia in 1984 [49]. This work and other more recent dose-ranging studies informed selection of PQ dosing in the present study [45, 49]. Risks of PQ administration have been further mitigated in the study with extensive G6PD screening using three modalities (rapid test, qualitative, and quantitative testing) on individuals aged 13 years and over who will receive PQ. Data generated by the study will be highly informative for defining the minimum diagnostic G6PD-deficiency screening requirements to deliver a safe and effective elimination program nationally.

### Vector interventions

Another component of the study is designed to measure whether using permethrin-treated clothing provides additional protection against malaria infection. The incremental benefit of an oft-proposed outdoor-biting vector intervention – permethrin-treated clothing – will be assessed. The results of limited prior clinical evidence suggested mixed outcomes. The intent of the present study is to evaluate potential additive benefits of permethrin-treated uniforms over the primary pharmacological interventions. Evidence for effectiveness of permethrin-treated clothing is largely limited to entomological studies given the scarcity of controlled clinical trials evaluating disease outcomes [50]. While the RCAF already provide long-lasting insecticide-treated nets (LLINs) to their soldiers, the effectiveness of this approach is unclear. The majority of malaria-transmitting mosquitoes in the region are thought to be outdoor, day-biting species although considerable heterogeneity in biting behaviors has been observed [51, 52]. Three small randomized, placebo-controlled studies of insecticide-treated clothing have demonstrated protection from malaria in similar settings, [50, 53, 54], but it remains unclear whether previous results showing benefit can be reproduced in the GMS, and if permethrin treatment is cost-effective. Additional vector control modalities, such as indoor residual spraying (IRS) and skin repellants, will not be included in the present study given the added expense of interventions, the nature of military service (mostly conducted outdoors), and the lack of added benefit demonstrated in areas with already high ITN coverage [55, 56].

### Limitations

In the present pilot study it is necessary to make trade-offs between complete rigor in statistical calculations in order to conduct the study among military personnel actively guarding a national border and their families. The study protocol aims not only to assess the comparative clinical effectiveness of MMP versus FSAT interventions (the primary objective), but also the practical feasibility of conducting intensive interventions at scale. A key deliverable to public health authorities will be a candid assessment of the degree to which competing elimination strategies (e.g., MMP versus FSAT effectiveness) could be achieved with limited medical supervision and safety monitoring in the field. As a result, clinical effectiveness and practical feasibility will be equally important outcome measures to determine next steps. It will be an ongoing challenge to conduct well-controlled studies in austere border areas, let alone rigorous elimination efforts. The study protocol design is logistically constrained by virtue of being primarily conducted in an active duty military population. The practical limits imposed on the number of clusters available, and finite follow-up periods are functions of the potential impact of the study on availability of RCAF personnel. While the limited number of clusters may effectively reduce power to draw definitive conclusions from the study, we accepted this compromise in order to assess operational feasibility. A randomized study of geographically isolated clusters also risks disease transmission from surrounding inhabitants and those choosing not to enroll in the study. This is another accepted risk. Because the present study cannot be confused with a public health directive by the government, it will be unethical to compel any inhabitant within or surrounding designated clusters to participate. This will be accounted for in the final interpretation of study results. The risks of transmission from unenrolled persons in the area will also be assessed by study team members during the trial (e.g., by documenting how many persons in a given area do not enroll) so as to incorporate into the final analysis to the best of our abilities.

Analysis of the effectiveness of MMP versus FSAT is the primary intervention (and outcome) of interest based on absolute risk reduction at 24 weeks. While the MMP arm reverts to standard-of-care (e.g., FSAT) after the 12-week intervention period, it would be unethical to not treat study participants who suffer new malaria infections during the follow-up period. This is intended to simulate a likely elimination program where an intervention is tried, and then the community defaults to standard of care while it is monitored afterward to determine effectiveness. While there is the potential for overlap between groups due to treatment during the follow-up period, the hypothesis is that the more intensive MMP intervention will reduce overall risk of disease further than the less intensive FSAT approach in the allotted study window. While we also wish to assess any additive effects of permethrin-treated clothing over pharmacological interventions in the treated clusters, the study is not powered based on permethrin treatment. In addition to estimation of additive effects, the intervention was performed to assess feasibility to effectively implement permethrin treatment and determine adherence to clothing wear.

It is anticipated and often argued that the large effort required to effectively achieve malaria elimination will preclude medical and safety monitoring. The feasibility of this approach will be assessed through analysis of diagnostic and adverse event data. If determined that elimination objectives could not be met without the intensity of medical monitoring to be employed here, it is possible that the study may conclude that approaches to malaria elimination studied here are not currently feasible. Technical, organizational and/or resource constraints may favor a more gradual approach as patient safety must always remain a paramount consideration. Regardless of outcomes, this initial study will serve as a pilot for scale-up efforts, and inform future operational approaches. Lastly, in an effort to optimize safety, due to phlebotomy limits for younger children, pediatric volunteers aged less than 13 years will not be screened for G6PD deficiency and will not receive PQ. While not expected based on known demographics of military dependents living along Cambodia’s borders, higher than anticipated pediatric enrollment could limit the study’s ability to discern potential benefits of the selected PQ dose.

### Translating results into action

As Cambodia approaches its stated goal of *P. falciparum* malaria elimination by 2020, and all malaria eliminated by 2025, a multitude of research efforts are underway to develop best practices. While mathematical models provide some guidance, evidence from randomized controlled trials on the most effective practical interventions for elimination is lacking. To date, most trials of mass drug administration have been historical, poorly designed, non-randomized or controlled [48]. Use of malaria prophylaxis has rarely been a stated objective of such studies beyond the secondary effects of mass treatment. The study will provide essential evidence to formulate a national elimination strategy serving vulnerable mobile populations who remain reservoirs for malaria transmission, comparing a large-scale prevention approach with current efforts to screen and treat individuals. At the same time, the study will strengthen the RCAF’s ability to pursue malaria elimination by strengthening surveillance capacity, managing information systems, and improving the quality of care. Ultimately the study will inform the Cambodian Government and other stakeholders regarding a practical approach to elimination in an underserved but critically important population.

## Additional file

Additional file 1: Standard Protocol Items: Recommendations for Interventional Trials (SPIRIT) 2013 Checklist: recommended items to address in a clinical trial protocol and related documents. (DOC 147 kb)

## Abbreviations

ACT: Artemisinin combination therapy
DHA-PIP: Dihydroartemisinin-piperaquine
FSAT: Focused screening and treating
G6PD: Glucose-6-phosphate dehydrogenase
LLINS: Long-lasting insecticidal nets
MMP: Monthly malaria prophylaxis
PPM: Personal protective measures
PQ: Primaquine
RDT: Rapid diagnostic test
WHO: World Health Organization
