# Supplementary material for: Cluster-randomized trial of monthly malaria prophylaxis versus focused screening and treatment: a study protocol to define malaria elimination strategies in Cambodia
Source: Trials. 2018 Oct 16;19:558. doi: 10.1186/s13063-018-2931-x (PMC6192281; doi:10.1186/s13063-018-2931-x)
Supplement: Supplementary file 1 — Standard Protocol Items: Recommendations for Interventional Trials (SPIRIT) 2013 Checklist: recommended items to address in a clinical trial protocol and related documents. (DOC 147 kb) [file 13063_2018_2931_MOESM1_ESM.doc]

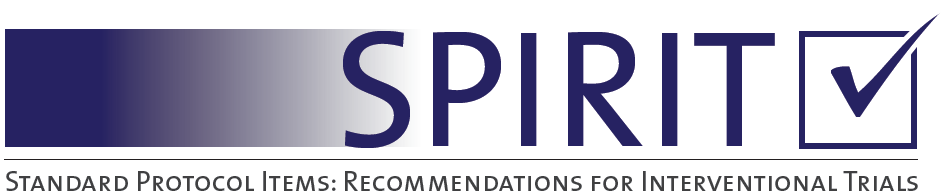


SPIRIT 2013 Checklist: Recommended items to address in a clinical trial protocol and related documents*

| Section/item | ItemNo | Description |
| --- | --- | --- |
| **Administrative information** | | |
| Title | 1 | | Descriptive title identifying the study design, population, interventions, and, if applicable, trial acronym | | --- |   Defining effective, appropriate, implementable strategies for malaria elimination in military forces in Cambodia as a model for mobile populations.  WRAIR Protocol Number: WRAIR # 2211  HRPO Log Number A-18805 |
| Trial registration | 2a | Trial identifier and registry name. If not yet registered, name of intended registry  NCT02653898 for Malaria Elimination Pilot Study in Military Forces in Cambodia |
|  | 2b | All items from the World Health Organization Trial Registration Data Set YES |
| Protocol version | 3 | Date Version 1.5, 22 June 2015 |
| Funding | 4 | Funding Source: Bill and Melinda Gates Foundation, Sponsor: USAMD- AFRIMS |
| Roles and responsibilities | 5a | Names, affiliations, and roles of protocol contributors:  **Principal Investigators:**  Chanthap Lon, M.D., M.P.H.M.; Armed Forces Research Institute of Medical Sciences (AFRIMS)  Sok Somethy M.A.; Ministry of National Defense  **Clinical Investigators:**  Dr. Muth Sinoun, MD, Deputy Director of CNM  David Saunders, M.D., M.P.H.; AFRIMS  Michele Spring, M.D., M.S.P.H; Military Malaria Research Program (MMRP), Walter Reed Army Institute of Research  Mariusz Wojnarski, M.D.; AFRIMS  Prom Satharath, M.D.; Ministry of National Defense, Cambodia  So Mary, M.D.; Chief Health Officer Region 4, Cambodia  **Clinical Research Coordinator:**  Soklyda Chann, RN; AFRIMS  **Laboratory Investigators:**  COL Philip Smith, Ph.D.; AFRIMS  MAJ Elizabeth Wanja, AFRIMS  MAJ Patrick McCardle; Div. of Entomology, WRAIR  Panita Gosi, PhD.; AFRIMS  Steven R Meshnick, MD, PhD; Jonathan Juliano, MD; Jessica Lin, MD  UNC School of Medicine, Chapel Hill, NC 27599  **Medical Monitor:** Kong Phan, M.A.; Anlong Veng Referral Hospital  **Laboratory Coordinators:**  Worachet Kuntawunginn; AFRIMS  Montri Arsanok; AFRIMS  **Ombudsmen:** Nou Sam On, Medical Assistant and  Mok Keng, Medical Assistant; Anlong Veng Referral Hospital  **Consultants:**  Dr. Huy Rekol, MD, Director, CNM  Dr. Lek Dysoley, MD, Deputy Director, CNM  Dr. Char Meng Chuor; Ministry of Health; Phnom Penh, Cambodia  Jessica Manning, M.D., M.Sc.; National Institutes of Health  Institutions engaged in Human Subjects Research:  National Center for Malaria, Parasitology and Entomology (CNM)  Ministry of National Defense, Royal Cambodian Armed Forces  Armed Forces Research Institute of Medical Sciences (AFRIMS) |
|  | 5b | Name and contact information for the trial sponsor:  The Armed Forces Research Institute of Medical Sciences (AFRIMS) was responsible for the overall conduct of the study as the sponsor-investigator in collaboration with other named investigating institutions engaged in human subjects research. |
|  | 5c | Role of study sponsor and funders, if any, in study design; collection, management, analysis, and interpretation of data; writing of the report; and the decision to submit the report for publication, including whether they will have ultimate authority over any of these activities  The Bill and Melinda Gates Foundation provided all funding for the study but had no role in study design, collection, management, analysis or interpretation of data; writing the report; or the decision to submit for publication. |
|  | 5d | Composition, roles, and responsibilities of the coordinating centre, steering committee, endpoint adjudication committee, data management team, and other individuals or groups overseeing the trial, if applicable (see Item 21a for data monitoring committee) N/A |
| Introduction |  |  |
| Background and rationale | 6a | Description of research question and justification for undertaking the trial, including summary of relevant studies (published and unpublished) examining benefits and harms for each intervention  Antimalarial drug resistance has reached critical levels on the Thai-Cambodian border. Many have begun advocating for concerted malaria elimination efforts in Cambodia. However, there is currently no consensus on how malaria elimination is to be achieved with the tools available.  In this study, the investigators will conduct operational research with the Royal Cambodian Armed Forces (RCAF) and National Malaria Center (CNM) to quantify the relative effectiveness of the two major interventional approaches - monthly malaria prophylaxis (MMP) or focused screening and treatment (FSAT) - in a head to-head comparison. In addition, the investigators will quantify the relative contribution of a recently advocated vector intervention for military personnel - the insecticide treated uniform (ITU) - in addition to other vector control measures currently employed by the RCAF. The investigators will employ the same permethrin insecticide self-application kits currently used by the US military. The investigators will estimate the cost effectiveness of each approach and attempt to define the best way forward for malaria elimination efforts in a critically important malaria reservoir in military population (and their dependents) who reside on the Thai-Cambodian border. The aim of the study is not only to conduct research to better define the best way forward in malaria elimination efforts in the high risk military populations, but to also build capacity within the RCAF to support and lead future elimination efforts in the most difficult-to-reach mobile populations. See clinical protocol background section for full details. |
|  | 6b | Explanation for choice of comparators  This is a two-arm, cluster-randomized, open-label controlled trial of monthly malaria prophylaxis (MMP) with three-day dihydroartemisinin-piperaquine (DHA-PIP) and weekly primaquine compared to focused screening and treating (FSAT) following current national treatment guidelines. See the study protocol background sections for full details. |
| Objectives | 7 | Specific objectives or hypotheses  To compare the effectiveness of focused screening and treatment (FSAT) following current national treatment guidelines versus monthly malaria prophylaxis (MMP) with dihydroartemisinin-piperaquine (DHA-PIP) in combination with weekly primaquine (PQ) (22.5mg) as transmission-blocking and radical curative agent to reduce the risk of malaria infection in personnel residing in military encampments on the Cambodian-Thai border. |
| Trial design | 8 | Description of trial design including type of trial (eg, parallel group, crossover, factorial, single group), allocation ratio, and framework (eg, superiority, equivalence, non-inferiority, exploratory)  This is a cluster-randomized, open label interventional study to determine the feasibility of achieving significant reduction in malaria cases in military encampments on the Thai-Cambodian border. The study will compare the effectiveness, safety, and tolerability of monthly malaria prophylaxis (MMP) to monthly focused screening and treatment (FSAT). This study will thus investigate the effectiveness of two potential interventions for malaria elimination. Subjects in the monthly malaria prophylaxis (MMP) arm will receive a standard 3-day treatment course of dihydroartemisinin-piperaquine on months 1, 2 and 3 and weekly low-dose primaquine (22.5mg for 12 weeks). Volunteers in the focused screening and treatment (FSAT) arm will be screened monthly and then treated for malaria following national treatment guidelines. For G6PD-deficient volunteers in the FSAT arm, primaquine will be administered weekly (45mg for 8 weeks) as radical curative and/or presumptive anti-relapse therapy. For G6PD normal volunteers with vivax infection, primaquine will be administered daily (15mg for 14 days). All FSAT volunteers with confirmed P. falciparum infection will receive a single, low dose (15mg) Primaquine as a P. falciparum transmission-blocking agent. The incremental benefit of an insecticide treated uniform (ITU) will also be assessed as a single-blind sham-controlled intervention in addition to personal protective measures currently employed by the RCAF. Volunteers will be followed monthly for a total of 6 months, to determine the proportion remaining malaria-free on day 180 following enrolment.  Refer to the clinical study protocol for full details. |
| Methods: Participants, interventions, and outcomes | | |
| Study setting | 9 | Description of study settings (eg, community clinic, academic hospital) and list of countries where data will be collected. Reference to where list of study sites can be obtained  The study is conducted in eight separate military encampments in Cambodia. |
| Eligibility criteria | 10 | Inclusion and exclusion criteria for participants. If applicable, eligibility criteria for study centres and individuals who will perform the interventions (eg, surgeons, psychotherapists)  Inclusion criteria:  1.Military volunteers aged 18-65 years of age plus their dependents > 2 years of age, eligible for care at an RCAF facility, or otherwise eligible Cambodian civilians at risk for contracting malaria who live within the designated geographical areas  2. Able to give informed consent/assent  3. Resides in the selected study areas, and available for monthly follow-up for 6 month study duration  4. Agrees not to seek outside medical care for febrile illness unless referred by study team  5. Authorized by local commander to participate in the study if on active duty  Exclusion criteria:  1.Allergic reaction or contraindication to dihydroartemisinin-piperaquine or primaquine or artesunate+mefloquine  2. Pregnant or lactating female, or female of childbearing age, up to 50 years of age or otherwise individually assessed for childbearing potential, who does not agree to use an acceptable form of contraception during the study  3.Judged by the investigator to be otherwise unsuitable for study participation |
| Interventions | 11a | Interventions for each group with sufficient detail to allow replication, including how and when they will be administered  See clinicaltrials.gov entry for a full description https://clinicaltrials.gov/ct2/show/NCT02653898 |
|  | 11b | Criteria for discontinuing or modifying allocated interventions for a given trial participant (eg, drug dose change in response to harms, participant request, or improving/worsening disease)  Any volunteer with G6PD deficiency who experiences grade 3 hemolysis following primaquine administration will be discontinued from the use of primaquine. Discontinued volunteers will be monitored closely according to protocol safety follow-up procedures, and treated with any necessary interventions as required for the volunteer’s safety.  If more than 3 subjects with G6PD deficiency are found to have grade 3 hemolysis following treatment with primaquine for anti-relapse therapy, further treatment with primaquine will be suspended for all G6PD deficient subjects enrolled in the study. |
|  | 11c | Strategies to improve adherence to intervention protocols, and any procedures for monitoring adherence (eg, drug tablet return, laboratory tests)  All subjects will be treated by directly observed therapy by a study team member. |
|  | 11d | Relevant concomitant care and interventions that are permitted or prohibited during the trial  Use of antimalarials or drugs with known antimalarial activity other than those prescribed by an investigator during the study will not be permitted. There are no other explicitly restricted concomitant medications for other disease during this study. |
| Outcomes | 12 | Primary, secondary, and other outcomes, including the specific measurement variable (eg, systolic blood pressure), analysis metric (eg, change from baseline, final value, time to event), method of aggregation (eg, median, proportion), and time point for each outcome. Explanation of the clinical relevance of chosen efficacy and harm outcomes is strongly recommended  Primary Outcome Measure:  The absolute risk reduction based on the proportion of subjects remaining malaria-free at the end of 6 months between the study arms as diagnosed by PCR-corrected malaria microscopy [Time Frame: 6 months ]  Secondary Outcome Measures :  Overall rate of sexual stage infections at Months 1 through 6 in each arm based on a combined endpoint of light microscopy and PCR analysis for detection of gametocyte maturity. [Time Frame: 6 months]  Number of participants with abnormal lab values and/or Adverse Events that are related to the treatments in each arm [ Time Frame: 6 months ]  Kaplan-Meier survival analysis of asexual and sexual blood stage at 28-day intervals after treatment or prophylaxis up to 180 days [ Time Frame: 6 months ]  Comparison of all-species and species-specific malaria incidence density in each arm over 180-day period [ Time Frame: 6 months ]  Comparative incidence of malaria detected by RDT versus RT-PCR versus microscopy [ Time Frame: 6 months ]  Comparative incidence of G6PD deficiency in the study population as determined by RDT, quantitative, and qualitative tests [ Time Frame: At the time of enrolment ]  Estimate of apparent rates of pre-existing immunity to malaria based on medical history, days of fever prior to presentation, and pre-existing parasitological parameters (gametocytemia, low asexual stage parasitemia) [ Time Frame: 6 months ]  Sensitivity and specificity assessment of the currently recommended rapid diagnostic test in Cambodia to detect moderate to severe G6PD deficiency using quantitative G6PD testing as the reference standard [ Time Frame: At the time of enrolment ] |
| Participant timeline | 13 | Time schedule of enrolment, interventions (including any run-ins and washouts), assessments, and visits for participants. A schematic diagram is highly recommended (see Figure)  See Manuscript Table 2 |
| Sample size | 14 | Estimated number of participants needed to achieve study objectives and how it was determined, including clinical and statistical assumptions supporting any sample size calculations  See Sample Size in Study Protocol. |
| Recruitment | 15 | Strategies for achieving adequate participant enrolment to reach target sample size  In addition to establishing good communications with community stakeholders, including relevant military commands, key messages will be disseminated prior to the study regarding the risks and benefits of participation. A video using an IRB-approved script will be used to describe the study in Khmer to potential enrolees. During the study, continuous outreach by study personnel will ensure maximum follow-up rates. |
| **Methods: Assignment of interventions (for controlled trials)** | | |
| Allocation: |  |  |
| Sequence generation | 16a | Method of generating the allocation sequence (eg, computer-generated random numbers), and list of any factors for stratification. To reduce predictability of a random sequence, details of any planned restriction (eg, blocking) should be provided in a separate document that is unavailable to those who enrol participants or assign interventions  Geographical areas will be randomized 1:1 allocation for MMP +/- vector control versus FSAT+/- vector control using time and region-blocked randomization to ensure that randomization is geographically interspersed. |
| Allocation concealment mechanism | 16b | Mechanism of implementing the allocation sequence (e.g., central telephone; sequentially numbered, opaque, sealed envelopes), describing any steps to conceal the sequence until interventions are assigned  Volunteers meeting enrolment criteria will receive permethrin-treated uniforms or sham uniforms according to the randomization at their geographically designated location in single-blind fashion (volunteer blinded to assignment but not investigator). There is otherwise no blinding to treatment allocation given geographic cluster design. |
| Implementation | 16c | Who will generate the allocation sequence, who will enrol participants, and who will assign participants to interventions  Principal Investigators generate the allocation sequence and coordinate with local commanders and medics at each site to ensure participants are appropriately assigned to interventions based on cluster designations.  Principle Investigators may delegate participant enrolment to study team members who satisfactorily complete training on protocol procedures, standard operating procedures, and subject consent and enrolment criteria. |
| Blinding (masking) | 17a | Who will be blinded after assignment to interventions (eg, trial participants, care providers, outcome assessors, data analysts), and how  Volunteers meeting enrolment criteria will receive permethrin-treated uniforms or sham uniforms according to the randomization at their geographically designated location in single-blind fashion (volunteer blinded to assignment but not investigator). See Section 6.7.1 which describes blinding of the insecticide-treated uniform (ITU).  Microscopists will be blinded to each other’s readings and to study arm assignment. There is otherwise no blinding during the study. |
|  | 17b | If blinded, circumstances under which unblinding is permissible, and procedure for revealing a participant’s allocated intervention during the trial  No unblinding is indicated based on study intervention. There should be little risk of inadvertent unblinding based on minimal potential differences in uniform odor immediately post treatment. |
| **Methods: Data collection, management, and analysis** | | |
| Data collection methods | 18a | Plans for assessment and collection of outcome, baseline, and other trial data, including any related processes to promote data quality (eg, duplicate measurements, training of assessors) and a description of study instruments (eg, questionnaires, laboratory tests) along with their reliability and validity, if known. Reference to where data collection forms can be found, if not in the protocol  See clinical protocol section 6.11 |
|  | 18b | Plans to promote participant retention and complete follow-up, including list of any outcome data to be collected for participants who discontinue or deviate from intervention protocols  Team-scheduled appointments for volunteers and home visits if the volunteers don’t return for a scheduled visit. |
| Data management | 19 | Plans for data entry, coding, security, and storage, including any related processes to promote data quality (eg, double data entry; range checks for data values). Reference to where details of data management procedures can be found, if not in the protocol  See Study Protocol Section 6.11 |
| Statistical methods | 20a | Statistical methods for analysing primary and secondary outcomes. Reference to where other details of the statistical analysis plan can be found, if not in the protocol  See Study Protocol Section 7 and 8 |
|  | 20b | Methods for any additional analyses (eg, subgroup and adjusted analyses)  See Study Protocol Sections 7 and 8 |
|  | 20c | Definition of analysis population relating to protocol non-adherence (eg, as randomised analysis), and any statistical methods to handle missing data (eg, multiple imputation)  See Study Protocol Sections 7 and 8 |
| **Methods: Monitoring** | | |
| Data monitoring | 21a | Composition of data monitoring committee (DMC); summary of its role and reporting structure; statement of whether it is independent from the sponsor and competing interests; and reference to where further details about its charter can be found, if not in the protocol. Alternatively, an explanation of why a DMC is not needed  N/A – the primary interventions are unblended, and there is no indication for a data monitoring committee |
|  | 21b | Description of any interim analyses and stopping guidelines, including who will have access to these interim results and make the final decision to terminate the trial  N/A - No interim analyses are planned |
| Harms | 22 | Plans for collecting, assessing, reporting, and managing solicited and spontaneously reported adverse events and other unintended effects of trial interventions or trial conduct.  See study protocol section 6.12 |
| Auditing | 23 | Frequency and procedures for auditing trial conduct, if any, and whether the process will be independent from investigators and the sponsor.  The US and Cambodian IRBs may audit the study at any time. The US IRB (WRAIR IRB) audited the study site in the year prior to study enrolment, and conducts annual visits to AFRIMS for the purposes of quality assurance. |
| Ethics and dissemination | | |
| Research ethics approval | 24 | Plans for seeking research ethics committee/institutional review board (REC/IRB) approval  The study protocol was approved by both IRBs prior to study start. |
| Protocol amendments | 25 | Plans for communicating important protocol modifications (eg, changes to eligibility criteria, outcomes, analyses) to relevant parties (eg, investigators, REC/IRBs, trial participants, trial registries, journals, regulators)  Important protocol modifications will be first approved by all relevant regulating and implementing authorities including all IRBs prior to implementation. Changes will be communicated with all study personnel prior to implementation, and if needed, with trial registries. Trial participants will be re-consented prior to continued participation. |
| Consent or assent | 26a | Who will obtain informed consent or assent from potential trial participants or authorised surrogates, and how (see Item 32)  Informed consent will be obtained by the Principal Investigators and designated study personnel as delegated by the PIs following satisfactory completion of appropriate training. |
|  | 26b | Additional consent provisions for collection and use of participant data and biological specimens in ancillary studies, if applicable  Volunteers will be consented for future storage and use of specimens at the time of enrolment if agreeable. |
| Confidentiality | 27 | How personal information about potential and enrolled participants will be collected, shared, and maintained in order to protect confidentiality before, during, and after the trial  All screening log contents of personal information that could link to volunteers’ charts and ICF were kept with security place that could access only study team with permission by PI.  IRBs will be able to access if requested. |
| Declaration of interests | 28 | Financial and other competing interests for principal investigators for the overall trial and each study site  Yes – all main study investigators declared the Conflict of interest prior to study initiated. |
| Access to data | 29 | Statement of who will have access to the final trial dataset, and disclosure of contractual agreements that limit such access for investigators  Only PIs and investigators. |
| Ancillary and post-trial care | 30 | Provisions, if any, for ancillary and post-trial care, and for compensation to those who suffer harm from trial participation  The volunteer will receive medical care for that injury at the appropriate medical facility, at no cost and volunteer receive their compensation per visit. |
| Dissemination policy | 31a | Plans for investigators and sponsor to communicate trial results to participants, healthcare professionals, the public, and other relevant groups (eg, via publication, reporting in results databases, or other data sharing arrangements), including any publication restrictions   - Report to RCAF and local commanders and medic. - Publication - Sharing with partners under permission by RCAF |
|  | 31b | Authorship eligibility guidelines and any intended use of professional writers  Yes |
|  | 31c | Plans, if any, for granting public access to the full protocol, participant-level dataset, and statistical code  Yes |
| Appendices |  |  |
| Informed consent materials | 32 | Model consent form and other related documentation given to participants and authorised surrogates  Video consent for group consent before individual consent. |
| Biological specimens | 33 | Plans for collection, laboratory evaluation, and storage of biological specimens for genetic or molecular analysis in the current trial and for future use in ancillary studies, if applicable.  Yes |

*It is strongly recommended that this checklist be read in conjunction with the SPIRIT 2013 Explanation & Elaboration for important clarification on the items. Amendments to the protocol should be tracked and dated. The SPIRIT checklist is copyrighted by the SPIRIT Group under the Creative Commons “[Attribution-NonCommercial-NoDerivs 3.0 Unported](http://www.creativecommons.org/licenses/by-nc-nd/3.0/)” license.
